# Public Involvement and Engagement in Big Data Research: Scoping Review

**DOI:** 10.2196/56673

**Published:** 2024-08-16

**Authors:** Piotr Teodorowski, Elisa Jones, Naheed Tahir, Saiqa Ahmed, Sarah E Rodgers, Lucy Frith

**Affiliations:** 1 Faculty of Health Sciences and Sport University of Stirling Stirling United Kingdom; 2 Department of Public Health, Policy & Systems University of Liverpool Liverpool United Kingdom; 3 National Institute for Health and Care Research Applied Research Collaboration North West Coast Liverpool United Kingdom; 4 Centre for Social Ethics and Policy University of Manchester Manchester United Kingdom

**Keywords:** patient and public involvement, PPI, involvement, engagement, big data, data science, patient engagement, co-design, coproduction

## Abstract

**Background:**

The success of big data initiatives depends on public support. Public involvement and engagement could be a way of establishing public support for big data research.

**Objective:**

This review aims to synthesize the evidence on public involvement and engagement in big data research.

**Methods:**

This scoping review mapped the current evidence on public involvement and engagement activities in big data research. We searched 5 electronic databases, followed by additional manual searches of Google Scholar and gray literature. In total, 2 public contributors were involved at all stages of the review.

**Results:**

A total of 53 papers were included in the scoping review. The review showed the ways in which the public could be involved and engaged in big data research. The papers discussed a broad range of involvement activities, who could be involved or engaged, and the importance of the context in which public involvement and engagement occur. The findings show how public involvement, engagement, and consultation could be delivered in big data research. Furthermore, the review provides examples of potential outcomes that were produced by involving and engaging the public in big data research.

**Conclusions:**

This review provides an overview of the current evidence on public involvement and engagement in big data research. While the evidence is mostly derived from discussion papers, it is still valuable in illustrating how public involvement and engagement in big data research can be implemented and what outcomes they may yield. Further research and evaluation of public involvement and engagement in big data research are needed to better understand how to effectively involve and engage the public in big data research.

**International Registered Report Identifier (IRRID):**

RR2-https://doi.org/10.1136/bmjopen-2021-050167

## Introduction

### Background

The growth of big data allows researchers to use and link large, multisource health data sets for research. Big data is still an evolving field [1], and disagreements remain on precisely what the term stands for in health research [2]. Other terms used include routinely collected data [3] and data-intensive research [1,4]. For clarity, throughout this paper, we will refer broadly to the term big data as it is used in the literature and easily understood by the public. We follow the definition by Aitken et al [1], recognizing that the main feature of big data is the ability to link large data sets for analysis. They name sources for such data as patient records, administrative, registry biobanking, social media, and digital application data. Big data research in health can be used for multiple purposes with the aim of improving health care services and reducing health inequalities [5,6]. These include service management, evaluation or audit of services, statistics, and exploring connections between health and non–health-related outcomes [1]. Often, these purposes differ from the original intent of data collection (eg, health care or statistical purposes). In other words, big data is often used for secondary research purposes.

Big data research offers new opportunities for academics. However, reusing big data for research faces ethical challenges [7]. Previous big data initiatives suggest that the public must have confidence that their data will be used in an acceptable way if they are going to be supportive of big data research [8]. This means moving outside what is legally required and establishing a social license for research [9]. Carter et al [9] proposed 3 conditions for establishing a social license for big data research. First, reciprocity is essential, as there is a need for 2-way communication and improving public awareness of big data research as well as improving researchers’ understanding of the public’s concerns and expectations. A lack of transparency could make it challenging to secure public trust [10], and the public has a right to be informed about the progress of the research [11]. Second, the process should empower, not disempower, the public; in big data research, this could include members of the public involved in the governance of data linkage and the design of big data projects. Third, big data research should benefit the public; thus, researchers need to understand what the public might perceive as public benefit.

Public involvement and engagement could be used to bridge the gap between researchers and the publics’ understandings of the benefits of big data research [12]. There is evidence in the literature (outside big data) that public involvement can provide legitimacy for research [13]. Public contributors could be a part of the process of creating research norms for big data research [14]. Research norms consist of governance and regulation that could guide research. These might not be popular among some academics, but they could help secure a social license for research [15]. Aitken et al [1], in their consensus statement on public involvement with big data research, go a step further and argue that “the public should not be characterised as a problem to be overcome but a key part of the solution to establish beneficial data-intensive health research for all.” There is emerging evidence that public contributors can be meaningfully involved in big data research projects [16-18]. However, there is a need to understand how public involvement and engagement takes place in big data research comprehensively.

### Objectives

Previous reviews have examined literature around public trust and attitudes toward big data research [19-22]. Despite public involvement and engagement being seen as one of the ways to improve public trust, as far as we are aware, there have not been any previous reviews exploring public involvement and engagement in big data research and there have not been any reviews registered on the PROSPERO and Cochrane databases. Therefore, this review aimed to synthesize what is known about public involvement and engagement in big data research. Using scoping review methodology [23-25], we mapped key issues in the research to find evidence of how public involvement and engagement were carried out in big data research. Understanding how to involve and engage the public in big data research could be used to formulate guidance for researchers and policy makers on how to do this effectively, as there are field-related challenges, especially regarding the abstraction and complexity of big data [26].

## Methods

### Overview

The protocol for this scoping stage review was published previously [27]. The protocol outlines the parameters of the review and provides a justification and explanation of all the methodological steps and decisions taken. To ensure rigor further, we used the PRISMA-ScR (Preferred Reporting Items for Systematic Reviews and Meta-Analyses extension for Scoping Reviews) checklist [28] and reported it as Multimedia Appendix 1.

### Defining Public Involvement

In the literature, the terms involvement, engagement, and participation are used interchangeably, but these do not always have the same meaning [29,30]. This makes research and discussion about public involvement challenging, as it can be difficult to identify papers for review [31-33]. Hence, there is growing recognition that more consistent terminology is needed [13]. The diversity of types of involvement can be seen in the ladder by Arnstein [34] that determines types of involvement by constructing a typology based on the amount of power given to the public. It identifies from the bottom (lowest extent of people’s influence) to the top (highest extent of people’s influence) the following steps: therapy manipulation, nonparticipation, informing, consultation, placation, partnership, delegation, and full citizen control. The author herself called the ladder “provocative.” One of the health-specific definitions of public involvement has been developed by INVOLVE [35]. It has been used broadly by funders and researchers and embedded in the public involvement reporting checklist [33]. It offers a nuanced perspective on 3 types of activities: involvement, engagement, and consultation, which researchers can use when working with members of the public. One is not better than the other, but rather, each offers a different approach. INVOLVE defines involvement as research carried out with or by members of the public rather than to, about, or for them. This recognizes shared ownership of research with members of the public. Engagement is providing information about big data research and disseminating it to the public. Consultation happens when the research is discussed with the public, but there is no shared ownership. Thus, engagement and consultation are “to,” “about,” or “for” rather than “with” or “by” them. However, these activities can provide an understanding of the public views.

Owing to the diversity of definitions of public involvement and engagement used in the literature, we mapped all included papers using the INVOLVE definition, identifying whether they were involvement, engagement, or consultation.

### Public Involvement in the Review

Public involvement in reviews can improve their quality by contributing to defining the scope, appraising the papers, and interpreting results [36,37]. In total, 2 public contributors (SA and NT) were involved in the review from the initial design stage and contributed at each stage (screening, data extraction, and analysis). They are both experienced public contributors and previously copublished papers around public involvement and engagement in big data research. SA and NT ensured the relevance of review results to the public. This was achieved by relating results to their experience as public contributors in other research projects. The details of the involvement process and what was put in place to support them (eg, training) are reported elsewhere.

### Searches

Following the search strategy developed with the support of a university librarian, the CINAHL, Health Research Premium Collection, PubMed, Scopus, and Web of Science databases were searched for papers in September 2021. The search strategy, as published in the protocol paper, is included in Multimedia Appendix 2. The search covered papers published after 2010 until the search completion in September 2021. Additional manual searches were conducted. These included the screening of the first 100 results from a Google Scholar search, journals that aim to publish public involvement research (*BMC Research Involvement and Engagement* and *Health Expectations*) or had special editions on public involvement in big data (*International Journal of Population Data Science*), and gray literature (the first 100 results from the Patient Outcome Research Institute database were screened). A call for potential papers to be included was posted on X (previously known as Twitter) to reach experts in the field.

### Inclusion Criteria

The review included papers that met the following criteria: (1) discussed public involvement or engagement in big data research (those that appeared more as consultations were not excluded, but a note was taken of this), (2) focused on patient- or health-related research, and (3) were published in English. All study designs and nonempirical discussion papers were included.

### Screening and Study Selection

PT took the lead by screening all papers. SA, NT, and EJ jointly screened at least a random 20% of papers at each stage (title, abstract, and full paper). Any discrepancies were discussed by the research team. The reasons for exclusions at a full paper stage were recorded and reported in the PRISMA (Preferred Reporting Items for Systematic Reviews and Meta-Analyses) checklist.

### Data Extraction

The data extraction form development was iterative and tested by the whole research team. The final data extraction form is available in Multimedia Appendix 3. PT extracted data from all papers in the first instance. Then, all extraction was double checked by the rest of the research team, thus ensuring each paper was considered by 2 researchers. The research team met regularly to discuss any discrepancies and discuss initial findings. PT organized the extracted data in a descriptive and narrative way under key headings based on the data extraction form. This was discussed with the research team.

### Analysis

**Figure 1:**
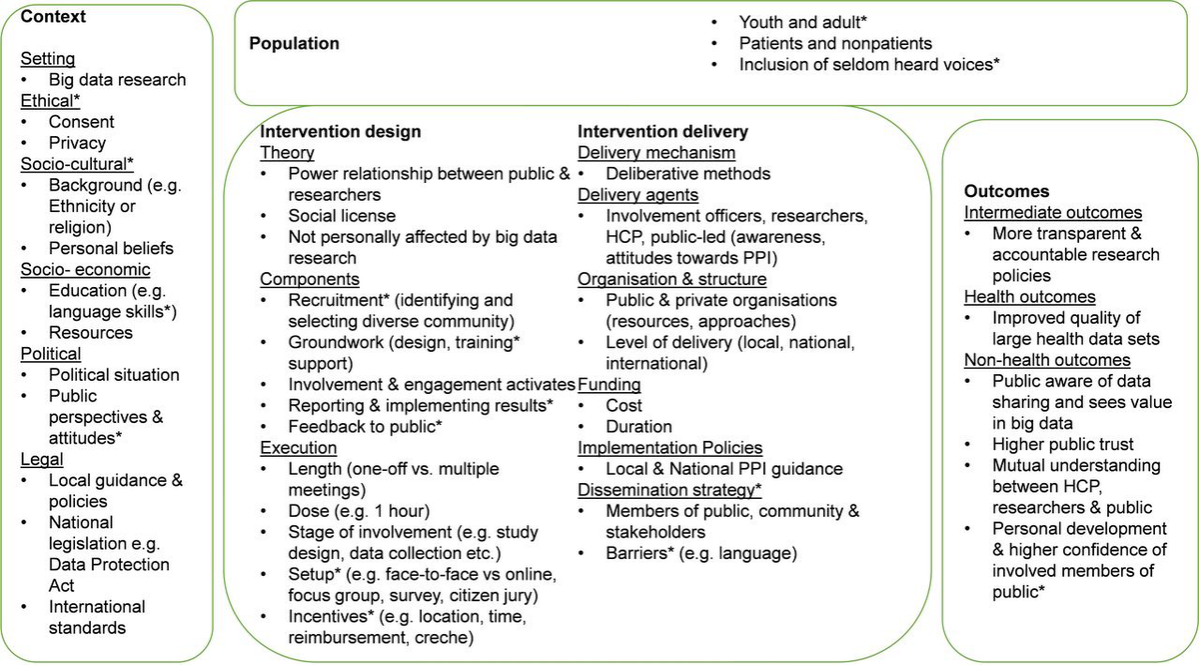
System logic model of public involvement and engagement in big data research (reproduced from the study by Teodorowski et al). HCP: health care provider; PPI: public and patient involvement.

The analysis was supported by a prior system logic model that we published in the protocol paper (Figure 1 [27]). It was initially developed by a preliminary scoping of the literature, research team discussion, and input from the public contributors. The logic model assisted us in identifying relevant elements of public involvement and engagement in big data research. We mapped our findings under the model and present them using headings from the logic model.

## Results

### Overview

**Figure 2:**
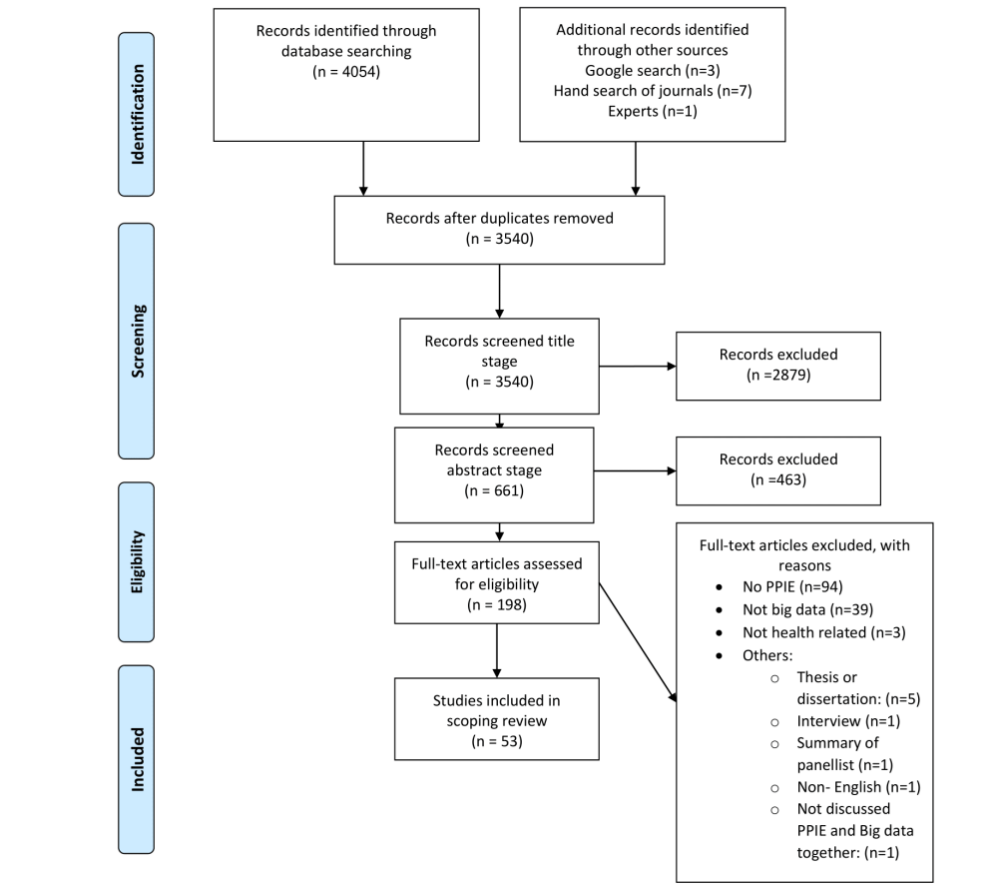
PRISMA (Preferred Reporting Items for Systematic Reviews and Meta-Analyses) flowchart. PPIE: patient and public involvement and engagement.

**Figure 3:**
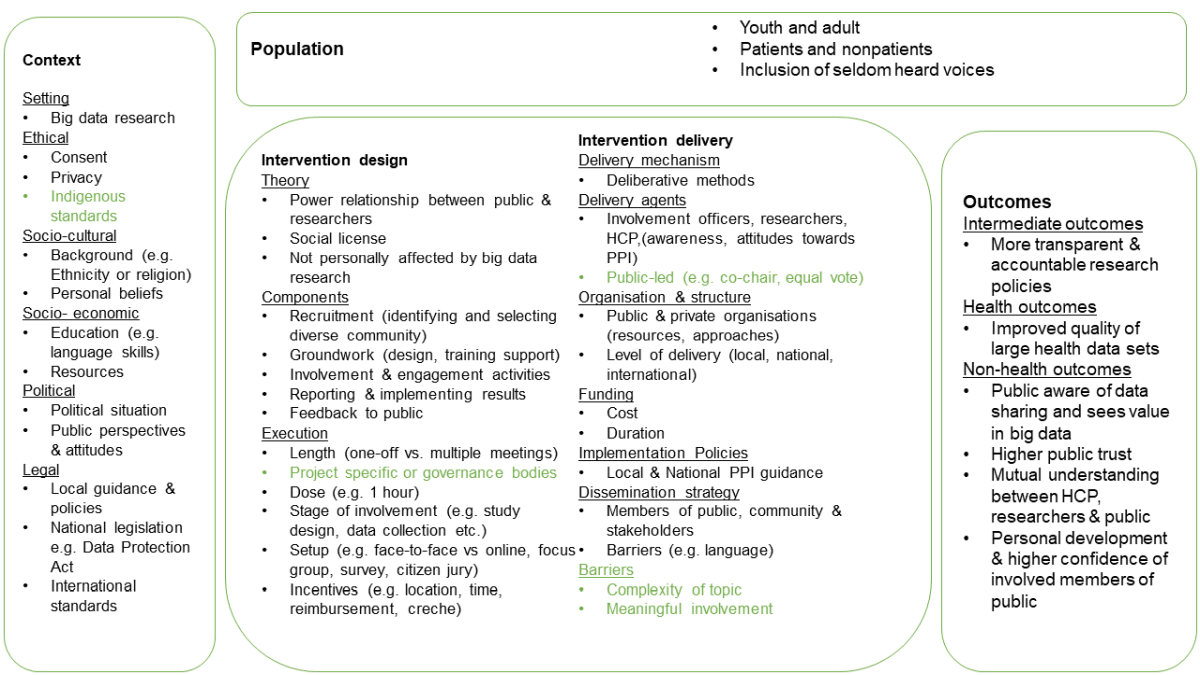
The updated a priori system logic model of public involvement and engagement in big data research (adapted from the study by Teodorowski et al). Green color is used to record new aspects of the model based on the review. HCP: health care provider; PPI: public and patient involvement.

The database searches produced 4054 papers. Additional manual searches added a further 11 papers. After the removal of duplicates, 3540 articles were screened for inclusion in the review. A total of 3342 papers were excluded based on the title and abstract. The full-text screen took place for 198 papers, and 53 were included in the review. Figure 2 [38,39] shows the PRISMA flowchart of the screening process. We first discuss the study characteristics and thereafter present findings as mapped under the revised system logic model (Figure 3 [27]).

### Study Characteristics

The most prevalent type of papers were discussion papers (nonempirical, including conceptual or ethical papers; 28/53, 53%), followed by review papers (5/53, 9%); qualitative study design (5/53, 9%); opinion, letter, commentary, or editorial (4/53, 8%); evaluation (3/53, 6%); protocol (2/53, 4%); ethnographic or descriptive case study (2/53, 4%); public deliberations (1/53, 2%); action research (1/53, 2%); quantitative (1/53, 2%); and mixed methods (1/53, 2%). The papers were from the United Kingdom (19/53, 36%), the United States (10/53, 19%), Canada (7/53, 13%), New Zealand (3/53, 6%), the Netherlands (1/53, 2%), Portugal (1/53, 2%), France (1/53, 2%), South Africa (1/53, 2%), Australia (1/53, 2%), Germany (1/53, 2%), and Africa (1/53, 2%). In total, 12 papers did not specify a geographical location, and some papers included more than one. The most prevalent type of involvement and engagement activities carried out with the public (following INVOLVE definitions) were involvement (45/53, 85%), followed by engagement (25/53, 47%) and consultation (7/53, 13%). Some papers discussed >1 type of activity. Table 1 presents the characteristics of the included papers.

**Table 1:** Summary of the included papers in the scoping review.

Study; year	Design	Location	Demographics to involve and engage	Types of activities	Area of interest
Baart and Abma [40], 2010	Action research	Netherlands	Not specified	Involvement and engagement	Involvement in psychiatric genomics research
Ballantyne and Style [41], 2017	Discussion	New Zealand	Lay, gender, and Māori representation	Involvement and engagement	Expert health data research ethics committee
Ballantyne and Stewart [42], 2019	Discussion	United Kingdom	Affected group; priority is given to patient groups considered vulnerable	Involvement and engagement	Public and private sectors collaborate to share, analyze, and use biomedical big data
Beyer et al [43], 2010	Qualitative	United States	Caucasian, Hispanic, Taidam or Lao; represented various education, income, and other characteristics	Involvement and consultation	Geocoded health information and experiential geographical information in a GIS^a^ environment
Bharti et al [44], 2021	Discussion	United Kingdom	Not specified	Engagement	Securing public trust and the importance of public engagement
Bot et al [45], 2019	Discussion	United States	Underrepresented populations	Involvement	Decentralization of governance
Coulter [46], 2021	Editorial	United Kingdom	General public	Involvement	National Health Services Digital plans to update its systems from patient data from general practitioner records
Dankar et al [47], 2018	Discussion	N/A^b^	Not specified	Engagement	Data governance in population genome projects
de Freitas et al [48], 2021	Protocol	Portugal	Patients and informal carers	Involvement	Coproduction of a people-centered model for the public in decision-making processes about data reuse
Deverka et al [49], 2019	Public deliberations	United States	Diverse geographic and individuals with chronic illness	Involvement and consultation	Recommendations for medical information commons design and management
Duchange et al [50], 2014	Discussion	France (European Union project)	Representatives of patient organizations	Involvement, engagement, and consultation	Ethics committee
Erikainen et al [51], 2020	Qualitative	United Kingdom	Not specified	Involvement	Governance of population-level biomedical research
Evans et al [52], 2020	Qualitative	United States	Individuals with OUD^c^ and their families	Involvement and engagement	Reuse of big data on opioid use
Fernando et al [53], 2019	Letter	South Africa	Traditional community leaders	Involvement and consultation	Data governance model in biobanking and data sharing
Fleurence et al [54], 2014	Discussion	United States	Patients	Involvement	National research network (PCORnet)
Funnell et al [55], 2020	Discussion	Canada	Indigenous communities	Involvement	Community-based participatory research methods in a project using previously collected data to examine end-of-life health care
Gallier et al [56], 2021	Discussion	United Kingdom	Not specified	Involvement and engagement	PIONEER infrastructure and data access processes
Goytia et al [57], 2018	Qualitative	United States	Patients	Involvement and engagement	Views on big data research
Henare et al [58], 2019	Opinion	New Zealand	Indigenous people	Involvement and engagement	Road map for neuroendocrine tumor research to reflect the values of Indigenous people
Hudson et al [59], 2020	Discussion	N/A	Indigenous population	Involvement	Indigenous communities’ views on the sharing of genomic data
Hurt et al [60], 2019	Discussion	United Kingdom	Not specified	Involvement and engagement	Design of HealthWise Wales
Jewell et al [61], 2019	Evaluation	United Kingdom	Service users and carers	Involvement	Advisory group
Jones et al [18], 2013	Evaluation	United Kingdom	Consumers; at least 1 representative from an ethnic minority group	Involvement	Consumer panel
Jones et al [17], 2019	Discussion	United Kingdom	Not specified	Involvement and engagement	SAIL Databank
Jones et al, 2020 [16]	Evaluation	United Kingdom	Inclusive of all ages, ethnic groups, cultures, socioeconomic levels, lifestyles, and other definable interests	Involvement and engagement	SAIL Databank and related population data science initiatives
Kalkman et al [62], 2019	Systematic review	N/A	N/A	Involvement and engagement	Ethical guidelines for principles and norms pertaining to data sharing
Kirkham et al [63], 2021	Qualitative	N/A	People with lived experience of mental illness and experience with data science or research methods	Involvement	Best practice checklist for use in mental health data science
Luna Puerta et al [64], 2020	Scoping review	N/A	N/A	Involvement	Reporting the impact of public involvement in biobanks
Manrique de Lara and Peláez-Ballestas [65], 2020	Narrative review	N/A	N/A	Involvement and engagement	Bioethical perspectives of big data
Milne et al [66], 2021	Discussion	United States and North America	Not specified	Involvement	Data trust model in the governance of biobanks
Milne and Brayne [67], 2020	Discussion	N/A	Not specified	Involvement	Data governance in dementia
Mourby et al [68], 2019	Discussion	United Kingdom	Not specified	Involvement and engagement	Obstacles preventing data linkage research from reaching its full potential
Murtagh et al [69], 2018	Ethnographic case study	United Kingdom	Participants of genomic studies	Involvement and engagement	Foundational principles of data sharing infrastructure
Nelson and Burns [70], 2020	Discussion	United Kingdom	Most affected communities by the research	Engagement	ADRC NI^d^ approach to public engagement
Newburn et al [3], 2020	Discussion	United Kingdom	Service users; 1 activity targeted ethnic minority groups	Involvement and engagement	Service user participation in a data linkage study
Nunn et al [71], 2021	Mixed methods	Australia	Not specified	Involvement	Involvement in genomic research
O’Doherty et al [72], 2011	Discussion	Canada	Groups considered historically disadvantaged	Involvement and engagement	Biobank governance and principles to form governance structures
O’Doherty et al [73], 2021	Commentary	N/A	Not specified	Involvement	Functions of good governance
Ohno-Machado et al [74], 2014	Discussion	United States	Patients	Involvement and consultation	Setting up of the pSCANNER^e^
Omar et al [75], 2020	Discussion	N/A	Not specified	Involvement, engagement, and consultation	European network of excellence for big data in prostate cancer
Paprica et al [76], 2020	Discussion	Canada	Communities facing long-standing inequalities that are affected by the research	Involvement and engagement	Establishment and operation of data trusts
Patel et al [77], 2021	Quantitative	United Kingdom	Not specified	Involvement	The use of remote consultation and prescribing of psychiatric medications
Pavlenko et al [78], 2020	Systematic review	N/A	N/A	Involvement	Governance in clinical data warehouses internationally
Rowe et al [79], 2021	Discussion	Canada, New Zealand, and United States	Indigenous people	Involvement	Principles for linking Indigenous population data
Shaw et al [11], 2020	Discussion	United States, Canada, and United Kingdom	General public and specific communities (eg, African Americans, Indigenous people, people with disabilities, and people living with homelessness)	Engagement	Social license for big data initiatives
Sleigh and Vayena [80], 2021	Descriptive case study	Germany and United Kingdom	General public	Engagement	Visual public engagement campaigns
Teng et al [81], 2019	Discussion	Canada	Not specified	Involvement	Public deliberation event on the data linkage and reuse for research
Tindana et al [82], 2015	Review	Africa	People affected by the research	Involvement, engagement, and consultation	Community engagement in biomedical and genomic research
Townson et al [83], 2020	Discussion	United Kingdom	Not specified	Involvement and engagement	A model of public involvement and engagement
Vayena and Blasimme [84], 2017	Discussion	N/A	Patients	Involvement	Models of informational control in data-intense health care and clinical research
Weich et al [85], 2018	Protocol	United Kingdom	Mental health users and carers and people with lived experiences; ensure diversity of age, gender, and ethnicity	Involvement	Spatial and temporal variation in the use, effectiveness, and cost of community treatment orders through the analysis of routine administrative data
Willison et al [86], 2019	Discussion	Canada	Patient representatives with diabetes including Francophone, immigrant, and Indigenous populations	Involvement	Governance model for health data repositories
Xafis and Labude [87], 2019	Discussion	N/A	Not specified	Involvement and engagement	Ethics framework for big data in health and research

^a^GIS: Geographic Information Systems.

^b^N/A: not applicable.

^c^OUD: opioid use disorder.

^d^ADRC NI: Administrative Data Research Centre Northern Ireland.

^e^pSCANNER: patient-centered Scalable National Network for Effectiveness Research.

### Population

The demographics of the public or communities involved and engaged in big data research were diverse. These included patients (including consumers and service users; 12/53, 23%); affected groups or groups considered vulnerable (8/53, 15%); Indigenous communities (6/53, 11%); articles focusing on specific characteristics (eg, gender, age, income, education, or geography; 5/53, 9%); carers (4/53, 8%); the general public (3/53, 6%); ethnic minority groups (3/53, 6%); patient representative or community leaders (3/53, 6%); and research study participants (1/53, 2%).

Deciding who should be on advisory boards, how they should be selected, and what their role should be remained a challenge for researchers [82]. An important issue was representativeness; advisory boards were unlikely to represent all the public views [66,69,87]. No single committee could represent all communities (because of their diversity) [58,76]. Identifying the relevant communities was seen to be difficult [82]. This created the challenge of ensuring legitimate group representation [72]. Advisory groups often did not reach a broader population [68]; hence, involvement and engagement need to move away from the “usual suspects” [16,18,66,76]. There was the risk that more vocal individuals could dominate the discussion [82]. Public contributors could be chosen arbitrarily, for example, based on personal contracts, and thus, the process might not be transparent to the public [72]. This could lead to involving financially and politically motivated [49] or well-connected contributors [42]. The way to overcome these issues could be to recruit public contributors from the study participants; for example, participants could elect their own representatives or a marketing company could conduct the recruitment [72,81].

### Context

Researchers should respect local and seldom-heard groups’ traditional structures and ethical perspectives. Papers focusing on Indigenous communities showed already existing governance mechanisms supporting research with these groups [59,79]. Researchers should incorporate Indigenous culture, for example, traditional ceremonies, when involving the community [58]. Formalized agreements with Indigenous organizations could improve the relationship with that community [55]. This more nuanced approach to big data research could assist researchers in establishing trust with Indigenous communities rather than merely convincing them that this is the right thing to do [59].

Political situations or public perspectives and attitudes could influence how and why members of the public get involved in big data research. Secrecy could be a challenge [11]. Organizations might not want to share controversial information, and private companies may argue that sharing it might be against their commercial interests [42]. Involvement and engagement could have the potential to improve public trust in big data research but not necessarily in the research institution [51]. There could be historic mistrust from underserved communities, for example, African Americans, Indigenous communities, and people living with homelessness [11]. There was no guarantee that it would always be possible to maintain public trust in big data research [67].

### Intervention Design

#### Theory

Respectful, ongoing, genuine, and nonhierarchical interaction between researchers and the public was seen as necessary to build trust [16,87]. Building a relationship could take time [82]. It included the coownership of research [55] and should concentrate on what the public wants to know [40]. The reciprocal relationship was illustrated by Newburn et al [3], who organized workshops during which they delivered training for members of the public on using social media and research methodology. A clear purpose for the activity leads to realistic expectations [16]. The starting point for involvement might not be about assuming an equal partnership but an exploration of power relationships [40]. Working in smaller groups gave more opportunities for every public contributor to share their opinion [81]. Decisions could be made through consensus [55,86]. However, Ballantyne and Stewart [42] recognized that there would always be disagreements and that all opinions cannot always be acted on; in that case, there might be a need for a clear explanation of why these voices were not included.

Conducting involvement and engagement activities did not mean that public values are incorporated into big data research [72]. Involvement could be tokenistic without effecting real change, but this still could offer some form of legitimacy to researchers and the research [72]. There was a need to ensure a balanced power relationship between public contributors and the research team [48]. When public contributors joined already ongoing research projects, they had limited scope for impact (eg, amendments might not be allowed); thus, their involvement might turn more into consultation [66,82]. Some researchers did not support involvement and would prefer a deficit engagement model where the members of the public were simply informed about the research [40]. Researchers should reflect on how to ensure balance in engagement. It could be about raising awareness of big data research and understanding that it should not be limited to an already agreed outcome but rather an ongoing dialogue [16,17,76]. Public involvement and engagement should take place before any data sharing occurs [11].

#### Recruitment

Various ways could be used to reach diverse audiences [75,86]. Recruitment of public contributors was mostly through already existing groups such as involvement groups (eg, Jewell et al [61] used an established involvement register that was open for service users and their families or carers), patient organizations [18,61,74,75,85], clinical sites [74], or recruitment via newsletter distributed among study participants [60,83]. Working with intermediaries (eg, charities or community leaders) could improve the reach as they can provide advice about public perspectives or can become gatekeepers [70,82]. Public contributors might be unclear on their role at the beginning [18]. Therefore, clear criteria for the public are needed [66]. Promoting involvement should focus on seeing it as a reciprocal opportunity with benefits for both researchers and public contributors [3]. The recruitment advertisement should include a description of the role and the required skills [61]. The full research protocol with all methodological details should be available on request [3]. There was a perceived need for a transparent process of selecting public contributors to avoid tokenism [49,73]. Candidates could be interviewed to identify individuals with team working skills and the ability to contribute outside their own health situation [18,86], as public contributors’ emotional connection to the research could be both an enabler or a barrier to their involvement [71].

Engagement is about reaching the broader public, especially around dissemination [62,68]. The engagement was mentioned alongside education, as it showed how findings from big data projects were shared with the community [52]. Educating the public could be seen as paternalistic, one directional, and top down; hence, there was a need for 2-way communication [11,86]. Researchers should share any discussion from governance groups with a broader public [11,56]. These could be a brief web-based report of findings and key recommendations [43].

#### Contribution

Public contributors had various roles in big data research. First, they contributed to specific research projects. In some papers, the public contributors were involved at all stages, from study design and identifying research questions to analysis and dissemination [3,48,52,53,55,57,61,65,82,85,87]. Public contributors also acted as coinvestigators in big data research projects [3].

The other role was around data governance. Public contributors (or representatives of patient organizations) could be involved in (joint) data governance to ensure that research was done ethically (in terms of public interest and sensitivity risk), for example, by advising, cofinding new solutions, or cocreating guidance and policy [16,18,41,42,45,54,56,58-60,62,64,66-69,72-78,86]. Working with the public could offer a lay perspective and ensure that data access and research were in the public interest, and thus, this was argued to potentially pave the way for establishing public trust [17,18,41,56,60,66,68].

One paper reported that public contributors who were members of governance bodies acted as big data advocates [16]. However, their voice should be of equal value as other stakeholders [49]. For example, if the group felt that a big data project did not have enough public input, they could assign a public contributor to support that particular work [86]. The governance bodies could also assist with engaging the general public (eg, by reviewing lay information) and guide the recruitment of new public contributors [16]. The influence of governance groups differs, and O’Doherty et al [72] recommended flexible governance that could evolve as big data research develops. Some papers argued that a one-size-fits-all solution might never work in big data research or for diverse communities [45,58,68,82]. Embedding involvement in the governance of big data research may require novel solutions [51].

The public should receive understandable and educational information on project outcomes [75]. Engagement activities should be proportional to the nature and size of the project around big data research [42]. Therefore, the way these engagement activities looked differed between the papers that were included. The public could be reached through engagement events [16,65]. Events were held with service users [3]. Researchers attended and supported events, for example, during the colorectal cancer awareness month [43]. Interactive elements (graphics, videos, etc) were used during exhibitions to raise public awareness [80].

The consultation approach consisted of surveys [50,75], informal small group meetings (eg, town hall meetings) [82], or qualitative research that aimed to capture the public perspective before setting up the project using that community data [53]. These included focus groups (eg, exploring patients’ approach to patient engagement in governance and prioritizing research questions) and interviews (eg, to understand public views toward privacy) [74].

In-person activities could be time restrictive and cost restrictive for some communities [74]. Public contributors might not be able to attend meetings, sometimes without warning because of personal circumstances (eg, health treatment, work, or family responsibilities) [3,81].

### Intervention Delivery

#### Delivery Mechanism

Involvement around governing big data research could also be conducted as a one-off deliberation event [49,81] or a Delphi study [63]. A one-off deliberation process could be particularly beneficial for contentious issues [73].

#### Delivery Agents

Governance groups could be chaired or cochaired by a public contributor, and most members of these groups could be members of the public [60,66,74,86]. If there was >1 governance group in the organization, public contributors could sit on different panels [16-18,74]. The public could be a part of the engagement process. Townson et al [83] mentioned the role of “Champions” who promoted studies in general practitioner surgeries, large public events (eg, food festivals) reaching schools, and support events organized by researchers. Another role they had was that of “supports.” Supports (similarly, to champions) were to promote the research, but it took the form of a pledge; this was more casual, with no formal training or evaluation and no reimbursement. However, both roles were voluntary, with no specific targets to reach [83].

Involvement and engagement should be led by team members experienced in organizing and running these activities [16,48,60,70,76]. Other researchers should dedicate time to these activities (and this time should be embedded in the workload) [16]. Research team members and facilitators should be trained in public involvement [60,81]. Access to specialist training on involvement and engagement should be provided to both staff and the public [16].

#### Organization and Structure

Using modern technology, researchers could create a registry or website where the public can see who had access to their data and for what purpose or receive newsletters [3,41,47,72]. Newburn et al [3] aimed to share their research on social media (Twitter and Facebook). Nationwide campaigns could explain the benefits of big data research [52,57,80]. This should be done in the language (eg, Indigenous) the public understands [58]. The public could be further reached through patient organizations [3,75], and researchers could share (yearly) updates jointly with them [50].

#### Funding

Expectations around monetary compensation should be established from the start [82]. These could include reimbursement for time [61,72,81,83], travel [81], and childcare expenses [3]. Researchers should provide lunch [3] and use venues that are easily accessible by public transport [3]. If public contributors are paid equally to professionals in governing bodies, this might improve their involvement [49].

#### Implementation Policies

A minority of papers directly referred to involvement or engagement guidance. These included the UK National Standards for Public Involvement [16,60,61], National Institute for Health and Care Research (NIHR) definitions of involvement and engagement [3,83], the GRIPP2 (Guidance for Reporting Involvement of Patients and the Public) checklist [3,61], the consensus statement on public involvement and engagement with data-intensive health research [16], an academic model guiding involvement [40], and local policies or principles [47,79].

Some papers mentioned legal documents to justify involvement and engagement. These include data protection legislation [16,67], government policies [41,45], and legislation or treaties around Indigenous communities’ rights [55,58].

#### Dissemination Strategy

Researchers should communicate clearly, in lay language and without jargon, to ensure transparency [18,49,76]. The examples included jargon-free graphics [80], tailoring academic research to lay audience [40], and postsession informal debrief [69]. When reaching the broader public, researchers should aim to deliver the message themselves rather than through the lens of media to provide more balanced information [3]. Public contributors should receive training introducing them to big data research [18,48,69,83,86]. The availability of good-quality information on big data underpins meaningful public involvement [75,87]. Explanations could include links to Wikipedia [83]. Researchers should send information before activities to give people time to reflect on it [83]. Public contributors might need extra time to consider their responses [16].

#### Barriers

Meaningfully including public contributors in the governance of big data projects could be challenging. Big data could be a complex topic, and it is difficult to find, involve, and engage public contributors with sufficient big data expertise [18,40,47,49,52,57,65]. Potential contributors might feel apprehensive about contributing to complex research if they do not understand the technical jargon [16,42]. This could be further compounded by language and cultural barriers between researchers and the public [82]. Public contributors should be offered training and additional support as required, especially with complicated topics [61,83]. Support needs to be person-centered and based on each individual’s skills and experience [83]. These could include short lectures, group discussions, and opportunities to ask questions [61,66]. For example, Teng et al [81] sent a booklet written by researchers in lay language on big data with a special focus on data collection, regulation, data sharing, and public concerns. Involving people with experience in research could be an alternative [69]. Kirkham et al [63] included public contributors with big data research experience. Still, they recognize that people with a better understanding of big data might have different views than the general public.

Public involvement should be a meaningful process. Included papers suggested several ways to ensure that members of the public would feel comfortable and able to share their views. Before meeting other stakeholders, public contributors could meet first together [48]. When commenting on a new aspect of research, public contributors were invited to comment first [86]. Some papers described the beginning of the involvement process [40,81]. In the study by Teng et al [81], during the first day of activities, presentations were made to provide some background on big data research for public contributors. These were from the perspective of patients and seldom-heard communities. These presentations were not neutral but opinionated to show diverse views on big data research.

### Outcomes

Some included papers in the review claimed that involvement and engagement should have clear outcomes. First, it could identify gaps in knowledge and priorities for research [70]. Second, it could align researchers’ and institutional perspectives of public interest with public views [44], for example, by bringing together charity workers, service providers, elected politicians, and members of the public [54,70]. Third, public contributors involved in governing bodies could have the effect of improving trust and accountability [84]. Fourth, improving public awareness of big data might democratize health research [62]. For example, Vayena and Blasimme [84] argued further that blending citizen science and participatory models could offer more democracy in governance.

However, measuring the impact of involvement and engagement in big data research was challenging [3,64,82,83]. A scoping review by Luna Puerta et al [64] recognized that there was no consensus about the objectives of public involvement in big data research, which undermines the ability to measure impact. Another review by Tindana et al [82] found that the papers included in their review on community engagement did not evaluate the effectiveness of engagement activities.

Engagement through genuine public debate could help demonstrate that the public sector could be a trustworthy steward of patient data [42]. This should include any negative comments toward the initiative; these should be publicly shared, and justification should be provided as to why their feedback was not implemented [42]. Dankar et al [47], when discussing biomedical databases, suggested that sharing research findings should include reaching individuals with personalized research results; these need to be valuable and benefit individuals (eg, they could go for health tests or make life changes that improve their health).

## Discussion

### Principal Findings

This scoping review provides an overview of how public involvement and engagement have been used in big data research or how it has been argued that it could be applied. This is the first review exploring this issue. The review has shown that the public can and, many articles argue, should be involved and engaged in big data research in terms of individual initiatives and data governance. However, the findings indicate that there is no one right way to involve and engage the public in big data research. Those responsible for working with the public should consider what type of activities are most relevant to their work and should use multiple approaches (involvement, engagement, and consultations) to reach different communities. Some papers suggested using modern technology when engaging the public (eg, through a website or digital newsletter). However, most included papers were not primary studies.

The review indicates that many believe that public involvement and engagement have the potential to improve public trust and accountability for big data initiatives. However, there is limited literature on how public involvement and engagement might influence it. Future research should attempt to measure the impact of involvement and engagement in securing social license for big data research with the broader public. The initial step to improve this situation could be to ensure reporting by using standardized reporting guidance for public involvement, such as GRIPP2 [88].

References to public involvement and engagement guidance or legal documents in the included papers were limited. The consensus statement on public involvement and engagement with data-intensive health research [1] is relatively new. However, INVOLVE (now incorporated into the NIHR) has been active in the United Kingdom since 1996. This indicates that many included papers replicate similar discussions around principles involving and engaging the public rather than referring to already established standards. However, more big data–specific guidance is being developed by the Public Engagement in Data Research Initiative in the United Kingdom [89].

The findings of this review indicate that some challenges are particularly relevant for involvement and engagement in big data research. However, the review has also shown that public involvement and engagement in big data research are not dissimilar to other research fields, as they share aspects of involving and engaging the public, such as working with seldom-heard communities and addressing power balance. This suggests that big data researchers could also use generic public involvement resources, such as the National Standards for Public Involvement in the United Kingdom [90].

The main challenge is that big data research is a complex topic. It might not be easy to explain it briefly (or in accessible language) to potential public contributors or the public. The papers offered some suggestions on how these barriers could be overcome. Researchers need to ensure that they allocate sufficient time and resources when discussing big data research with members of the public. This finding aligns with another review that examined patient involvement in cancer research, where the authors identified time-consuming involvement as a primary challenge in that context [91]. This review suggests that involving and engaging the public in big data research might be even more time consuming than in other fields. If these challenges are overcome, there is a higher chance that involvement and engagement in big data research is not tokenistic, but this might mean additional time and financial resources. Researchers should budget for these resources as they design any involvement or engagement activities. However, they should be supported to do it by research institutions and funders.

Bailey et al [92] reported that Black and South Asian communities in the United Kingdom have less trust in the health system, and because of this, there might be concerns within these groups about how the public bodies use their data. Researchers need to recognize how trust and attitudes toward big data research could influence public involvement and engagement. This review has offered some indication of how to achieve this from the literature that explored working with Indigenous communities, such as recognizing communities’ beliefs and way of life.

The protocol that this review was based on presented the priori system logic model for public involvement and engagement in big data research [27]. On the basis of the review findings, the model was revised. Within the context section, Indigenous standards were added to recognize that big data research needs to consider the perspective and views of Indigenous communities that might differ from previous dominant perspectives. In the intervention theory section, the execution of involvement activities could be divided into project-specific aspects (eg, focusing on 1 big data research project) and governance bodies that look into granting approvals into data linkage (for other projects). These 2 purposes might influence how researchers involve and engage the public. In intervention delivery, the bullet point around public-led activities was added, as some papers suggested that it was important to ensure that the public voice is equivalent to professionals’ views during voting and should have equal or even more influence (eg, by cochairing meetings or being coinvestigators). Furthermore, a new bullet point was added in intervention delivery to recognize big data–specific barriers, especially jargon, and how complex big data research could be to members of the public.

Most of the elements included in the model were discussed in the included papers. The only exception is that it does not reflect on the involvement and engagement of people who are not personally affected by big data research (or do not perceive themselves as such). The coverage of most of the issues raised in the papers for involvement and engagement in big data research suggests that the logic model could support researchers who intend to design and deliver these activities to the public.

Textbox 1 provides a summary of the key recommendations around public involvement and engagement in big data research based on the review findings.

**Key recommendations around public involvement and engagement in big data research.**

- Ensure that complex and abstract language is explained in lay terms and is understandable to members of the public.
- As public involvement and engagement in big data research might require additional time and resources, these should be planned and budgeted in research plans.
- Trust and public attitudes could influence how and if members of the public get involved in big data research. Public involvement and engagement activities targeting seldom-heard communities should recognize the cultural beliefs held by these groups.
- Following big data research standards could provide researchers with more specific guidance for working with members of the public. These should be used alongside already existing generic guidance.
- Capture and evaluate the impact of public involvement and engagement activities in big data research.

### Limitations

The first limitation is the use of terminology. The review explored public involvement and engagement in big data research. These terms are used in different ways by researchers. This parallels the experience of Brett et al [93] in their review, where they found that the variability in wording used to describe involvement complicated literature searching. The search strategy was developed with an experienced librarian and included additional manual searches. However, this did not guarantee that all relevant papers were included. This could have influenced the search results, as potentially some relevant papers might not have been picked up by the search as the authors used different terms. The second limitation was that only information included in the papers was extracted. The authors of included papers were not approached for more details. As academic papers have a word limit, it is possible that some additional information about involvement and engagement may have not been included in the published paper. In contrast to the initial plan, the references of included papers were not screened for potential inclusion. This was because screening of references of included papers in the scoping review was considered impractical because of the high number of papers. Moreover, only papers published in English were included. Finally, owing to the number of papers identified through the searches, only a random sample of 20% was screened by all coauthors.

### Conclusions

This review offers a snapshot of evidence on what public involvement and engagement in big data research could look like. It is limited, as it was largely based on discussion papers, but useful, as evidence on how these involvement and engagement activities could be delivered and what type of outcomes they could produce was provided. The field would benefit from further research and evaluation of involvement and engagement activities in big data through primary research. Owing to the ongoing development of big data research, it is likely that these would need to be updated on a regular basis, but nevertheless, such research could provide further insights into how to meaningfully involve and engage the public in big data research.

## Appendix

Multimedia Appendix 1 PRISMA-ScR (Preferred Reporting Items for Systematic Reviews and Meta-Analyses extension for Scoping Reviews) checklist.

Multimedia Appendix 2 Search strategy as published in the protocol paper.

Multimedia Appendix 3 Data extraction form.

## Abbreviations

GRIPP2: Guidance for Reporting Involvement of Patients and the Public
NIHR: National Institute for Health and Care Research
PRISMA: Preferred Reporting Items for Systematic Reviews and Meta-Analyses
PRISMA-ScR: Preferred Reporting Items for Systematic Reviews and Meta-Analyses extension for Scoping Reviews
