# Supplementary material for: Public Involvement and Engagement in Big Data Research: Scoping Review
Source: J Particip Med. 2024 Aug 16;16:e56673. doi: 10.2196/56673 (PMC11364952; doi:10.2196/56673)
Supplement: Multimedia Appendix 2 [file jopm_v16i1e56673_app2.docx]

**Appendix 2**

Search strategy as published in the protocol paper.

| Public | “advisory group” OR carer* OR citizen* OR client* OR communit* OR consumer* OR famil* OR lay OR nonpatient* OR participant* OR patient* OR public OR relative* OR representative* OR stakeholder* OR “steering group*” OR survivor* OR user* |
| --- | --- |
| Involvement or engagement | advocacy OR collaborat* OR co*production OR consult* OR empower* OR engage* evaluat* OR involv* OR particip* OR partner* OR PPI OR organi*ation* OR representation* |
| Big data | database OR “big data" OR “data science” OR “data mining” OR “datasets” OR “data analytics” OR “data sets” |
| Public Involvement | “patient participation” OR “consumer participation” OR “client participation” OR “community participation” |
| Data governance (only Health Research Premium Collection, Scopus & Web of Science) | “data governance” |
