# Supplementary material for: Public Involvement and Engagement in Big Data Research: Scoping Review
Source: J Particip Med. 2024 Aug 16;16:e56673. doi: 10.2196/56673 (PMC11364952; doi:10.2196/56673)
Supplement: Multimedia Appendix 3 [file jopm_v16i1e56673_app3.docx]

**Appendix 3**

## **Data extraction form**

Title:

Year:

Author:

Number:

**Generic Information**

| Paper aim |  |
| --- | --- |
| Design (e.g. qualitative, discussion paper) |  |
| Country (if specified) |  |
| Context (e.g. health condition) |  |
| Demographics of participants for PPI (also record if there are a seldom-heard group based if paper defines them as such). |  |
| Methods (if relevant) |  |
| Funding |  |

**PPIE**

| Is It involvement, engagement or consultation (based on NIHR definition)? |  |
| --- | --- |
| Process of involvement or engagement (e.g. advisory board, co-researchers etc.) |  |
| Legal or ethical issues (in relation to PPI) |  |
| References to PPI guidance and policies |  |
| Challenges of PPI |  |
| Facilitators of PPI |  |
| Key Message |  |
| Other relevant information |  |
